# Lack of Clinically Significant Relationships of Age or Body Mass Index with Merkel Cell Carcinoma Immunotherapy Outcomes

**DOI:** 10.3390/cancers16132480

**Published:** 2024-07-07

**Authors:** Rian Alam, Xinyi Fan, Daniel S. Hippe, Lisa M. Tachiki, Emily Gong, Emily Huynh, Paul Nghiem, Song Youn Park

**Affiliations:** 1 Department of Dermatology, University of Washington, Seattle, WA 98109, USA; rianalam@uw.edu (R.A.);; 2 Fred Hutchinson Cancer Center, Clinical Research Division, Seattle, WA 98109, USA; 3 Division of Hematology/Oncology, Department of Medicine, University of Washington, Seattle, WA 98109, USA; 4 College of Osteopathic Medicine, Pacific Northwest University of Health Sciences, Yakima, WA 98901, USA

**Keywords:** Merkel cell carcinoma, immunotherapy, body mass index, age

## Abstract

**Simple Summary:**

Merkel cell carcinoma is a rare, aggressive skin cancer prone to spreading. While anti-PD-1/PD-L1 immunotherapy has improved outcomes for advanced MCC, about half of such patients do not see long-lasting benefits. This study examined how age and body mass index (BMI) affect the response to immunotherapy in 183 advanced MCC patients from a single-center-based, longitudinal database. The analysis showed that age impacts treatment response, with patients significantly older than 70 years responding less effectively. However, age did not influence other outcomes, including progression-free survival, MCC-specific survival, and overall survival. Similarly, BMI did not significantly affect any immunotherapy outcomes. These results suggest that age and BMI should not be used to determine immunotherapy eligibility in MCC, highlighting the need for unbiased patient selection for this treatment.

**Abstract:**

Merkel cell carcinoma (MCC) is a rare and aggressive skin cancer with a high risk of metastasis. The development of anti-PD-1/PD-L1 immunotherapy has improved outcomes for advanced MCC, yet about 50% of such patients do not achieve durable responses. This study analyzed the effects of age and body mass index (BMI) on immunotherapy response in 183 advanced MCC patients from a single-center longitudinal database. Using Fine–Gray or Cox regression models, treatment response, progression-free survival (PFS), MCC-specific survival, and overall survival (OS) were evaluated. Age showed a significant non-linear relationship with treatment response (*p* = 0.04), with patients much older or younger than 70 years less likely to respond. However, age was not significantly associated with PFS (*p* = 0.21), MCC-specific survival (*p* = 0.72), or OS (*p* = 0.36). Similarly, BMI was not significantly correlated with treatment response (*p* = 0.41), PFS (*p* = 0.52), MCC-specific survival (*p* = 0.78), or OS (*p* = 0.71). Unlike previous studies suggesting that obesity and advanced age improve outcomes in other cancers, these associations were not observed in MCC. These findings suggest that age and BMI should not influence eligibility for immunotherapy in MCC patients, emphasizing the importance of unbiased patient selection for this treatment.

## 1. Introduction

Merkel cell carcinoma (MCC) is an aggressive skin cancer with a rising incidence, predominantly affecting older adults [1,2]. Approximately 40% of patients are diagnosed at an advanced stage, and 40% of MCC patients experience recurrences following standard treatment, often necessitating systemic interventions [3].

Immunotherapy has emerged as the preferred first-line treatment for advanced-stage MCC [4]. Compared to chemotherapy, the previous standard of care, anti-PD-1/PD-L1 immunotherapy offers a comparable response rate of ~50%, vastly improved durability of response, and a favorable toxicity profile [5,6,7,8,9,10,11]. Despite the success of immunotherapy in MCC, more than 50% of patients do not persistently benefit from PD-1/PD-L1 immunotherapies [6,12].

As immunotherapy has become the dominant approach for treating advanced disease across various cancers, multiple studies have sought to identify factors influencing immunotherapy efficacy [13]. Research in other cancers, including melanoma, lung cancer, and renal cell carcinoma has suggested an association between age or body mass index (BMI) and immunotherapy response [14,15,16,17,18]. In this study, we investigated how age and BMI may correlate with immunotherapy response in MCC, utilizing a large longitudinal database of MCC patients.

## 2. Methods

### 2.1. Patient Selection

An IRB-approved, Seattle-based repository of MCC patients who enrolled between 2012 and 2021 was analyzed, retrospectively. This longitudinal database contains relevant clinical features and long-term follow-up information, and it is updated at least annually with information extracted from documented patient communication and medical records. Informed consent was obtained from all subjects involved in the study.

Only patients enrolled in the repository ≤180 days from the start of immunotherapy and prior to disease progression were included in this study to avoid ascertainment bias toward patients with worse outcomes. Patients who had histologically confirmed unresectable stage III or IV MCC (American Joint Committee on Cancer 8th Edition [19]) received first-line treatment with PD-1/L-1 immunotherapy, and had follow-up information available for response, progression, and survival outcomes. Clinical data collected at time of immunotherapy initiation included age, BMI, sex, disease stage, immunosuppression status, and ECOG performance status (Eastern Cooperative Oncology Group). BMI calculations at the start date of immunotherapy followed the National Institute of Health guidelines, computed as the ratio of weight in kilograms divided by the square of height in meters. Patient age was determined as years between date of birth and initiation of immunotherapy.

### 2.2. Outcome Measurements

Tumor response was classified as either progressive disease (PD), stable disease (SD), partial response (PR), or complete response (CR) in accordance with the Response Evaluation Criteria In Solid Tumors (RECIST version 1.1).

Time to objective response was determined as the time from immunotherapy initiation to the first time a CR or PR was documented, with death or progressive disease considered as competing risks. Progression-free survival (PFS) was defined as the time from immunotherapy initiation to either progressive disease, recurrence, or death. MCC-specific survival was defined as the time from immunotherapy initiation to patient death by MCC, with death from other causes considered as a competing risk. Overall survival (OS) was defined as the time from immunotherapy initiation to patient death due to all causes.

### 2.3. Statistical Considerations

All analyses were conducted using R (version 4.3.2, R Foundation for Statistical Computing). All statistical tests were two-sided and statistical significance was defined as *p* < 0.05. Associations of age and BMI with each outcome after immunotherapy were assessed using Fine–Gray regression models for outcomes with competing risks (objective response and MCC-specific survival) and Cox regression models for outcomes without competing risks (PFS and OS). Multivariable models included age, BMI, ECOG performance status (0 vs. 1+), gender, stage of disease (III vs. IV), and immunosuppression. In the primary analysis, age and BMI were treated as continuous variables, as opposed to being divided into discrete categories, to maximize statistical power and avoid threshold-dependent results. Potential non-linear relationships of age or BMI with outcomes were examined using restricted cubic splines with three knots. Knots were placed by default at the 10th, 50th (median), and 90th percentiles of the age or BMI distribution. The regression coefficient corresponding to non-linear portion of the restricted cubic spline was tested against zero using a Wald test. Relationships between age or BMI and each outcome were modeled as linear unless the test for a non-linear component was statistically significant. As a sensitivity analysis and to facilitate comparisons with other studies which categorized these variables, the analyses of age and BMI versus outcomes were repeated but with both factors categorized in different ways. Age was either categorized into four groups (<60, 60–69, 70–79, and ≥80 years) [20] or two groups (≤75 and >75 years) [21]. BMI was dichotomized two ways, either as ≥25 kg/m^2^ vs. <25 kg/m^2^ [17] or ≥30 kg/m^2^ vs. <30 kg/m^2^ [21].

## 3. Results

### 3.1. Clinical Characteristics of Cohort

A total of 183 patients were identified as eligible, with 140 male patients (76.5%) and 43 female patients (23.5%), with a median age of 70 (range: 19 to 91). Among the 169 patients (92%) with available BMI data, the median BMI was 30.0 kg/m^2^ (range: 19.4 to 58.7 kg/m^2^). A total of 33 patients had a BMI between 18.5 and 24.9 kg/m^2^ (19.5%), 61 patients with a BMI between 25 and 29.9 kg/m^2^ (36.1%), and 75 patients with BMI ≥ 30 kg/m^2^ (44.4%). A total of 167 patients had ECOG status 0 or 1 (93.3%). A total of 91 patients (51.1%) were seropositive for MCPyV oncoprotein antibody, 87 patients (48.9%) were seronegative, and 5 patients had no data available. There were 67 patients with unresectable stage III disease (36.6%) and 116 patients with stage IV disease (63.4%). Thirty-six patients (19.7%) had chronic immunosuppression. The age distribution in immunosuppressed and immunocompetent patients were similar, with both groups having a median age of 70 years (*p* = 0.64). The percentage of immunosuppression was slightly higher in the <60 age group (7/26, 27%) compared to the others (29/157, 18%) but this was not statistically significant (*p* = 0.30). Within this cohort, 106 patients (58.0%) received pembrolizumab, 54 patients received avelumab (29.5%), 20 patients (10.9%) received nivolumab, and 3 patients (1.6%) received nivolumab + ipilimumab. Detailed clinical features are summarized in Table 1.

### 3.2. Outcomes

The median follow-up time for this cohort was 27 months (range 0.7–81.6). Overall, the objective response rate at 6 months was 59.16% (95% CI: 51.47, 66.04). Estimated PFS, OS, and MCC-specific survival with 95% CI at 2 years were 53.03% (45.9, 61.23), 68.39% (61.58, 75.94), and 27.32% (20.69%, 34.35), respectively. The median time to objective response was 6.5 months. The median PFS and OS times were 29.6 months and 67 months, respectively, while the median MCC-specific survival time was not reached in this cohort.

### 3.3. Association of Age with Outcomes

**Figure 1:**
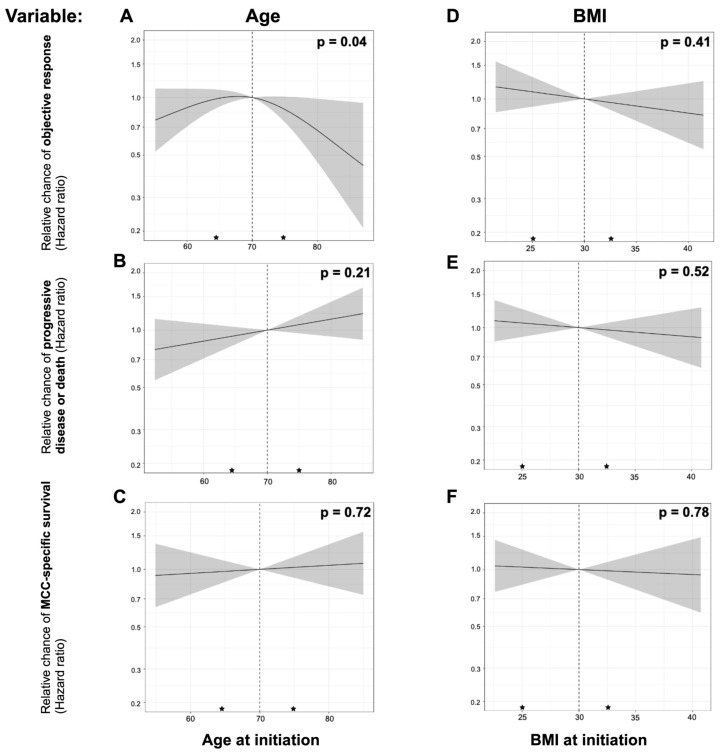
Impact of age and body mass index on immunotherapy outcomes relative to the median. In this cohort, progression-free survival (PFS) was defined as the time from IMTX initiation to either progressive disease, recurrence, or death. Objective response was determined as either CR or PR from the time of IMTX initiation. Age was treated as a non-linear variable for objective response, but as a linear variable for PFS and MCC-specific survival. Body mass index (BMI), Eastern Cooperative Oncology Group (ECOG) score, gender, stage, and immunosuppression status were all accounted for. The gray shaded area indicates the confidence interval. The median age was 70, with a first and third quartile value of 64 and 75, respectively. The median BMI was 30, with a first and third quartile value of 25.9 and 32.7, respectively. Black stars have been used to indicate quartile values, with the black dotted lines representing the medians. (**A**) shows that patients younger or older than the median age of 70 are less likely to achieve objective responses (*p* = 0.04). (**B**) shows that age is not significantly correlated with progression-free survival (*p* = 0.21). (**C**) also shows that age is not significantly correlated with MCC-specific survival (*p* = 0.72). (**D**–**F**) show that BMI is not significantly correlated with objective response, progression-free survival, or MCC-specific survival (*p* = 0.41, 0.52, and 0.78, respectively). CR = complete response; PR = partial response; IMTX = immunotherapy.

Associations between age and each outcome are summarized in Table 2 and Figure 1. Age had a statistically significant, non-linear association with objective response (*p* = 0.04), where patients much older or younger than the median age of 70 were less likely to respond to immunotherapy than patients around age 70. This decreased likelihood of response was more pronounced for older patients, where 80-year-old patients had a 33% reduced rate of response compared to 70-year-old patients (HR = 0.67, 95% CI: 0.46–0.99%, *p* = 0.04). Sixty-year-old patients had an 11% reduced rate of response compared to 70-year-old patients (HR = 0.89, 95% CI: 0.71–1.11, *p* = 0.30), though this decrease did not reach statistical significance.

By contrast, age was not statistically significantly associated with PFS (HR: 1.14 per 10-year increase, 95% CI: 0.93,1.41, *p* = 0.21), MCC-specific survival (HR:1.05 per 10-year increase, 95% CI: 0.80, 1.37, *p* = 0.72), or OS (HR: 1.12 per 10-year increase, 95% CI: 0.88, 1.42, *p* = 0.36, Table 2).

As a sensitivity analysis to facilitate comparisons with other studies, analyses with age were repeated with age categorized into four groups (<60, 60–69, 70–79, and ≥80 years) or two groups (≤75 and >75 years) and are summarized in Supplemental Table S1. When age was categorized as four groups, the 60–69 year (HR: 2.08, 95% CI: 1.10, 3.95, *p* = 0.025) and 70–79 year groups (HR: 1.62, 95% CI: 0.85, 3.10, *p* = 0.14) tended to have a high rate of objective response than the <60 year group. The <60 year group and ≥80 year groups had similar objective responses rates (HR: 1.02 [ref: <60 years], 95% CI: 0.43, 2.42, *p* = 0.96).

### 3.4. Association of BMI with Outcomes

Associations between BMI and each outcome are summarized in Table 2 and Figure 1. BMI was not statistically significantly associated with objective response (HR: 1.09 per 5 kg/m^2^ increase, 95% CI: 0.89, 1.33, *p* = 0.41), PFS (HR: 0.95 per 5 kg/m^2^ increase, 95% CI: 0.80, 1.12, *p* = 0.52), MCC-specific survival (HR: 0.97 per 5 kg/m^2^ increase, 95% CI: 0.79, 1.20, *p* = 0.78), or OS (HR: 0.96 per 5 kg/m^2^ increase, 95% CI (0.79,1.17), *p* = 0.71).

In sensitivity analyses, BMI was dichotomized as BMI ≥ 25 kg/m^2^ vs. BMI < 25 kg/m^2^ and BMI ≥ 30 kg/m^2^ vs. BMI < 30 kg/m^2^, results of which are summarized in Supplemental Table S2. In these additional analyses, BMI was not significantly associated with any of the four outcomes when dichotomized as BMI ≥ 25 kg/m^2^ (*p* = 0.46–0.75) or when dichotomized as BMI ≥ 30 kg/m^2^ (*p* = 0.38–0.82).

## 4. Discussion

PD-1/PD-L1 immunotherapy represents a significant advancement in cancer therapy [22]. Notably, in MCC over 50% of patients experience an objective response to immunotherapy. Numerous studies have explored clinical characteristics and biomarkers that may be predictive of immunotherapy response in MCC, although strongly predictive markers have proven elusive [23,24]. Prior studies have identified advanced age and higher BMI as being associated with a higher likelihood of immunotherapy response in several different types of solid tumors. The underlying mechanisms for these associations are not well understood. Notably, these associations have not been thoroughly examined in the context of MCC, a cancer that is particularly responsive to immunotherapy.

In our analysis, BMI did not show a significant correlation with immunotherapy outcomes in MCC. Conversely, age demonstrated a significant association with the rate of objective response; older individuals were found to be less likely to achieve an objective response, though age did not significantly affect other outcomes like PFS, OS, or disease-specific survival.

### 4.1. Age and PD-1/PD-L1 Immunotherapy Response and Long-Term Outcomes

Previous studies in melanoma, renal cell carcinoma, and non-small cell lung cancer have indicated improved immunotherapy outcomes associated with advancing age [16,20,25]. As reported in Kugel et al. [14], melanoma patients with age ≥62 had better responses to anti-PD-1 therapy. However, this association was relatively weak, with the odds of response only increasing by 13% per 10-year increase in age. This modest increase in response rate with age is consistent with the small increase we observed up to age 70 in our cohort of MCC patients. However, we also observed a more rapid decrease in response rate after age 70 (HR = 0.67 for age 80 vs. age 70, *p* = 0.04), which differs substantially from the melanoma cohort.

In a study by Lichenstein et al. [20] investigating non-small cell lung cancer among 245 patients, it was found that patients between 70 and 79 years had a significantly lower hazard for disease progression or death (hazard ratio: 0.60, *p* = 0.015) than younger patients, though PFS tended to be worse for patients ≥ 80 years old (HR: 1.62, *p* = 0.098).

In our cohort, we did not observe significantly better PFS for patients 70–79 vs. <60 (HR: 1.06, 95% CI: 0.54–2.07, *p* = 0.86, Supplemental Table S1), though the confidence interval includes the HR of 0.60 that Lichtenstein et al. [20] estimated. Our results for patients aged 80 and older were quite similar to Lichtenstein’s findings (HR: 1.89, *p* = 0.11).

Prior studies have suspected various potential mechanisms that relate to immune changes associated with age [26,27]. A recent study noted that aging is associated with increased tumor mutational burden, decreased promoter methylation and elevated expression of immune checkpoint genes, potentially leading to greater efficacy of immune checkpoint targeted therapies [28]. However, no definitive mechanism has been clearly elucidated. Further research should be conducted to investigate the various factors associated with aging that may potentially impact immunotherapy responses.

### 4.2. BMI and PD-1/PD-L1 Immunotherapy Response and Long-Term Outcomes

In several solid cancer types, a positive correlation has been observed between higher BMI and immunotherapy response [17,21,29,30]. In a study of renal cell carcinoma, higher BMI was associated with a small improvement in OS and PFS in two large cohorts (n = 1975 and 4657, HR: 0.81–0.86 for BMI ≥ 25 kg/m^2^ vs. <25 kg/m^2^) [17]. Another retrospective study by Wang et al. [18] involving 250 cancer patients, including those with lung cancer, ovarian cancer, or melanoma, found that obesity (BMI ≥ 30 kg/m^2^) was associated with improved progression-free survival (HR: 0.61, 95% CI: 0.42–0.89, *p* = 0.01) and overall survival (HR: 0.59, 95% CI: 0.35–0.99, *p* = 0.048) after adjusting for other risk factors. Similarly, a recent small (n = 32) European study by Incorvaia et al. [21] examined the impact of BMI on MCC immunotherapy outcomes and found that a BMI ≥ 30 kg/m^2^ was associated with significantly lower likelihood of MCC recurrence compared to a BMI < 30 kg/m^2^ (HR: 0.18, *p* = 0.04).

However, our cohort experienced a much smaller association of BMI ≥ 30 kg/m^2^ with MCC recurrence following IMTX (HR: 0.94, 95% CI: 0.62–1.44, *p* = 0.79) as compared to that of Incorvaia et al. [21]. Our confidence interval strongly excludes a hypothesis of HR = 0.18 from the Incorvaia study but only marginally excludes a hypothesis of HR = 0.61 from the Wang study. Both our study and the Incorvaia study are specific to MCC, but there are notable differences in the cohorts. The Incorvaia study included only patients treated with avelumab, while our study included patients treated with pembrolizumab, nivolumab, and avelumab. The median age in the Incorvaia study was 75, compared to 70 in our study, suggesting that age-related differences are unlikely to significantly affect the results. Our study may better reflect real-world experiences as the cohort size is six times larger and includes patients treated with both PD-1 and PD-L1 immunotherapy.

It should be acknowledged that BMI has limitations as an obesity assessment tool due to its inability to differentiate between adipose tissue and muscle [31]. Further investigation is needed to better understand the relationship between higher BMI and its effects on immune function, ideally using techniques that differentiate adipose tissue from muscle. Future research may also focus on linking obesity with changes in the gut microbiota. The composition of the gut microbiota, such as the quantity and diversity of said bacterium, has already been linked to play an important role in modulating responses to immunotherapy treatment [32].

### 4.3. Study Limitations

Limitations of this study include its retrospective nature and the relatively small cohort size, a consequence of the rarity of this cancer. However, to the best of our knowledge, this study represents the largest investigation into the potential correlation between BMI or age and immunotherapy response in MCC. 

## 5. Conclusions

Our study shows no clinically significant relationship between age, BMI, and response to IMTX for MCC. With 183 patients, we may have not been able to detect small associations between age or BMI and outcomes. However, the 95% confidence intervals for the corresponding hazard ratios suggest that any associations, if present, are likely not substantial enough to impact clinical decision-making. Our findings strongly indicate that older age and increased BMI should not discourage the use of immunotherapy as a treatment option and underscore the continued viability of PD1/PD-L1 immunotherapy as a first-line treatment for advanced MCC.

## Figures and Tables

**Table 1:** Patient demographics and clinical status at start of immunotherapy.

Variable	No.	%
Gender		
Female	43	23.5
Male	140	76.5
Age in years		
Median = 70 (range 19–91)		
<60	26	14.2
60–70	60	32.8
70–80	72	39.3
80+	25	13.7
BMI (kg/m^2^)		
Median = 30.0 (range 19.4–58.7)		
18.5–24.9	33	19.5
25–29.9	61	36.1
30+	75	44.4
Data not available	14	
ECOG performance status		
0	114	63.7
1	53	29.6
2	11	6.1
3	1	0.6
Data not available	4	
MCPyV Oncoprotein Antibody Status		
Seropositive	91	51.1
Seronegative	87	48.9
No data	5	
Stage		
III	67	36.6
IV	116	63.4
Immunosuppression type		
Immunocompetent	147	80.3
Immunosuppressed	36	19.7

BMI = body mass index; ECOG = Eastern Cooperative Oncology Group; MCPyV = Merkel cell polyomavirus.

**Table 2:** Associations of age and body mass index (BMI) with outcomes during immunotherapy using Cox or Fine–Grey regression model.

		Univariable		Multivariable *	
Predictor	Outcome	HR (95% CI) †	*p*-Value	HR (95% CI) †	*p*-Value
Age					
	Objective response ‡	Non-linear	0.04	Non-linear	0.04
	Progression-free survival	1.13 (0.92, 1.40)	0.24	1.14 (0.93, 1.41)	0.21
	MCC-specific survival	1.06 (0.81, 1.39)	0.66	1.05 (0.80, 1.37)	0.72
	Overall survival	1.15 (0.91, 1.46)	0.25	1.12 (0.88, 1.42)	0.36
BMI					
	Objective response	1.08 (0.91, 1.29)	0.37	1.09 (0.89, 1.33)	0.41
	Progression-free survival	0.97 (0.81, 1.15)	0.70	0.95 (0.80, 1.12)	0.52
	MCC-specific survival	0.99 (0.80, 1.23)	0.92	0.97 (0.79, 1.20)	0.78
	Overall survival	0.99 (0.80, 1.21)	0.90	0.96 (0.79, 1.17)	0.71

BMI = body mass index; HR = hazard ratio; CI = confidence interval; ECOG = Eastern Cooperative Oncology Group. * Multivariable models include age, BMI, gender, stage, immunosuppression status, and ECOG performance status. † The hazard ratios correspond to change in event rate per 10-year increase in age or change in event rate per 5 kg/m^2^ increase in BMI. ‡ A non-linear relationship was detected between age and objective response, which is characterized in Figure 1.

## Data Availability

The data presented in this study are available in this article and Supplementary Materials.

## Supplementary Materials

The following supporting information can be downloaded at: https://www.mdpi.com/article/10.3390/cancers16132480/s1, Table S1: Multivariable associations of age with outcomes after grouping age in different ways; Table S2: Multivariable associations of body mass index (BMI) with outcomes after grouping age in different ways.
