# Supplementary material for: Lack of Clinically Significant Relationships of Age or Body Mass Index with Merkel Cell Carcinoma Immunotherapy Outcomes
Source: Cancers (Basel). 2024 Jul 7;16(13):2480. doi: 10.3390/cancers16132480 (PMC11240446; doi:10.3390/cancers16132480)
Supplement: Supplementary file 1 [file cancers-16-02480-s001.zip › cancers-3083708-supplementary.pdf]

Supplemental Table S1. Multivariable associations* of age with outcomes after grouping age in different ways.

	Objective Response		Progression-free Survival		MCC-specific Survival		Overall Survival	
**Variable**	**HR (95% CI)**	**p-value**	**HR (95% CI)**	**p-value**	**HR (95% CI)**	**p-value**	**HR (95% CI)**	**p-value**
Age								
<60	(ref)		(ref)		(ref)		(ref)	
60-69	2.08 (1.10-3.95)	0.03	1.11 (0.55-2.24)	0.78	0.76 (0.33-1.76)	0.52	0.83 (0.36-1.93)	0.67
70-79	1.62 (0.85-3.10)	0.14	1.06 (0.54-2.07)	0.86	0.87 (0.40-1.91)	0.74	1.23 (0.57-2.62)	0.60
≥80	1.02 (0.43-2.42)	0.96	1.89 (0.87-4.10)	0.11	1.21 (0.49-3.00)	0.68	1.45 (0.59-3.61)	0.42
Age								
≤75 years	(ref)		(ref)		(ref)		(ref)	
>75 years	0.73 (0.46-1.17)	0.19	1.37 (0.84-2.23)	0.21	0.97 (0.51-1.83)	0.92	1.05 (0.59-1.86)	0.88

HR = hazard ratio; CI = confidence interval; BMI = body mass index; ECOG = Eastern Cooperative Oncology Group;

*All models include age (categorized into four groups or into two groups), BMI, gender, stage, immunosuppression status, and ECOG performance status.

Supplemental Table S2. Multivariable associations* of body mass index (BMI) with outcomes after grouping age in different ways.

Variable	Objective Response		Progression-free Survival		MCC-specific Survival		Overall Survival	
	HR (95% CI)	p-value	HR (95% CI)	p-value	HR (95% CI)	p-value	HR (95% CI)	p-value
BMI								
<25 kg/m^2	(ref)		(ref)		(ref)		(ref)	
≥25 kg/m^2	1.20 (0.73-1.97)	0.48	0.82 (0.49-1.38)	0.46	0.80 (0.41-1.55)	0.50	0.90 (0.49-1.67)	0.75
BMI								
<30 kg/m^2	(ref)		(ref)		(ref)		(ref)	
≥30 kg/m^2	1.18 (0.81-1.73)	0.38	0.94 (0.62-1.44)	0.79	0.84 (0.49-1.45)	0.54	0.94 (0.57-1.56)	0.82

HR = hazard ratio; CI = confidence interval; BMI = body mass index; ECOG = Eastern Cooperative Oncology Group;

*All models include age, BMI (dichotomized at 25 kg/m^2 or 30 kg/m^2), gender, stage, immunosuppression status, and ECOG performance status.
